# Transcriptome characterization of candidate genes for heat tolerance in perennial ryegrass after exogenous methyl Jasmonate application

**DOI:** 10.1186/s12870-021-03412-9

**Published:** 2022-02-12

**Authors:** Gang Nie, Jie Zhou, Yiwei Jiang, Jie He, Yang Wang, Zongchao Liao, Charlotte Appiah, Dandan Li, Guangyan Feng, Linkai Huang, Xia Wang, Xinquan Zhang

**Affiliations:** 1 Department of Forage Breeding and Cultivation, College of Grassland Science and Technology, Sichuan Agricultural University, Chengdu, 611130 China; 2 Department of Agronomy, Purdue University, West Lafayette, IN 47907 USA

**Keywords:** *Lolium perenne* L., Heat stress, MeJA, Chlorophyll, HSF-HSP network

## Abstract

Methyl jasmonate (MeJA) plays a role in improving plant stress tolerance. The molecular mechanisms associated with heat tolerance mediated by MeJA are not fully understood in perennial grass species. The study was designed to explore transcriptomic mechanisms underlying heat tolerance by exogenous MeJA in perennial ryegrass (*Lolium perenne* L.) using RNA-seq. Transcriptomic profiling was performed on plants under normal temperature (CK), high temperature for 12 h (H), MeJA pretreatment (T), MeJA pretreatment + H (T-H), respectively. The analysis of differentially expressed genes (DEGs) showed that H resulted in the most DEGs and T had the least, compared with CK. Among them, the DEGs related to the response to oxygen-containing compound was higher in CKvsH, while many genes related to photosynthetic system were down-regulated. The DEGs related to plastid components was higher in CKvsT. GO and KEGG analysis showed that exogenous application of MeJA enriched photosynthesis related pathways under heat stress. Exogenous MeJA significantly increased the expression of genes involved in chlorophyll (Chl) biosynthesis and antioxidant metabolism, and decreased the expression of Chl degradation genes, as well as the expression of heat shock transcription factor - heat shock protein (HSF-HSP) network under heat stress. The results indicated that exogenous application of MeJA improved the heat tolerance of perennial ryegrass by mediating expression of genes in different pathways, such as Chl biosynthesis and degradation, antioxidant enzyme system, HSF-HSP network and JAs biosynthesis.

**Supplementary Information:**

The online version contains supplementary material available at 10.1186/s12870-021-03412-9.

## Background

Perennial ryegrass (*Lolium perenne* L.) is a widely cultivated cool-season grass species in temperate regions. This species has high nutritive values and herbage yield, making it a suitable grass for forage production, while its fast establishment and good traffic tolerance are desirable for turfgrass uses [1, 2]. In addition, perennial ryegrass provides benefits on ecosystems such as promoting carbon assimilation, soil protection, and nutrient cycling [3]. However, perennial ryegrass is generally heat-sensitive, and high temperature is a major factor limiting its performance and utilization in many areas including temperate regions where a periodic heat stress can occur during the summertime [4, 5]. Thus, a better understanding of heat tolerance mechanisms will help to improve grass management and facilitate breeding programs for creating heat tolerant varieties of perennial ryegrass.

Heat stress can adversely affect growth and physiology of the plants. A number of physiological parameters, such as photochemical efficiency, photosynthetic rate, water use efficiency, and chlorophyll (Chl) content are often used as indicators for heat stress tolerance [6–8]. Leaf senescence, characterized by loss of Chl, is a hallmark of damage induced by heat stress among cool-season grass species. It was reported that heat-induced leaf senescence was attributed to heat accelerated Chl catabolism rather than attenuated Chl biosynthesis [9]. Furthermore, reduction of Chl catabolic rate by suppressing a Chl catabolic gene (pheophytin pheophorbide hydrolase, *PPH*) was found to delay heat-induced leaf senescence in perennial ryegrass [10], suggesting that Chl metabolism is crucial for heat tolerance. A study of evaluating 98 accessions of perennial ryegrass in response to heat stress showed that Chl content had the highest correlation with heat tolerance [11], indicating that Chl was the most closely linked parameter to heat tolerance. Maintaining the adequate carbohydrates including soluble sugars and fructan could effectively delay the speed of leaf senescence by protecting photosynthetic apparatus from heat damage and maintaining osmotic homeostasis and membrane stability in perennial ryegrass [12]. Collectively, heat induced leaf senescence involves key physiological changes in perennial grass species.

Plant hormones play a crucial role in adaptation to environmental stress. The higher levels of phytohormones such as abscisic acid, gibberellic acid, indole-3-acetic acid and zeatin riboside contribute to heat tolerance of perennial ryegrass [12]. Methylation jasmonic acid (MeJA) is a kind of jasmonic acid (JA) derivatives and can act as an endogenous signal molecule. Previous studies have shown that MeJA have a wide range of effects on plant development and abiotic tolerance [13, 14]. When plant is injured, the amount of MeJA content increases significantly, which stimulates the biosynthesis of some metabolites such as hormone and proline and induces the expression of genes related to stress tolerance [15]. Exogenous application of MeJA protected cell membranes from heat stress and improved the basal heat resistance of *Arabidopsis* [16]. In perennial ryegrass, MeJA-induced heat tolerance was involved in the maintenance of relative water content and Chl content as well as lower electrolyte leakage and malondialdehyde content [17]. For a plant cell to survive under heat stress, it is important to prevent protein folding and aggregation [18]. Heat shock proteins (HSPs) are considered as stress proteins, with a function of maintaining internal cell stability and protecting the injured organisms by enhancing the stability of mRNA and translation process under heat stress [19]. MeJA induced *HSP72* expression through *HSFI* activation in C6 glioma cells, thereby enabling animal cells to acquire the ability to respond to heat shock [20]. In tomato (*Lycopersicon esculentum* M.) fruits, MeJA induced the accumulation of sHSP and HSP70 transcripts, and the induced synthesis of HSPs may be involved in the protection of fruit from chilling injury [21]. HSP70 and HSP90 might act together after MeJA treatment to promote protection and maintenance of homeostasis within three hours of heat stress in opium poppy (*Papaver somniferum* L.) [22].

Although previous evidence indicated important roles of MeJA in regulating abiotic stress responses in plants, the molecular function of MeJA in relation to heat tolerance remains unclear in perennial grass species. In this study, Illumina NovaSeq platform was employed for transcriptome characterization of candidate genes related to heat tolerance in perennial ryegrass following exogenous MeJA application. The results would elucidate the possible MeJA-mediated mechanisms of heat tolerance in perennial grass species.

## Material and methods

### Plant materials and treatments

The perennial ryegrass cultivar ‘Esquire’ seeds were provided by DLF SEED A/S Company in China office. Seeds were germinated in plastic pots (20 cm length, 15 cm width, 10 cm height) filled with quartz sand and distilled water. The pots were placed in a growth chamber with temperatures of 20 °C /15 °C (day / night), 70% relative humidity, and photosynthetic active radiation of 750 μmol·m^2^·s^− 1^. After 7 days of germination, plants were irrigated with Hoagland nutrient solution and grew for another 30 days (Pots positions were rearranged daily to reduce the impact of the environment on plant growth). Divided the plant into two parts and 30 mL of 100 μM MeJA solution or H_2_O was applied three times (every other day) in 7-day period under 20 °C /15 °C. At the end of 7 days, half of the plants in each treatment remained under 20 °C, while the other half were subjected to heat stress at 38 °C for 12 h. The four treatments included: 1) control (CK), without MeJA pretreatment and heat stress; 2) Only MeJA pretreatment (T); 3) Only 38 °C heat stress (H); 4) MeJA pretreatment and heat stress (T-H). Leave samples were collected after 12 h of all treatments with three biological replicates, and then immediately stored in a − 80 °C refrigerator.

### RNA extraction and Illumina sequencing

Total RNA was extracted using Trizol reagent kit (Invitrogen, Carlsbad, CA, USA) according to the manufacturer’s protocol. RNA quality was assessed by Agilent 2100 Bioanalyzer (Agilent Technologies, Palo Alto, CA, USA) with the sample RNA integrity number (RIN) greater than 7, and checked using RNase free agarose gel electrophoresis. Eukaryotic mRNA was enriched by Oligo (dT) beads, while prokaryotic mRNA was enriched by removing rRNA by Ribo-ZeroTM Magnetic Kit (Epicentre, Madison, WI, USA). The enriched mRNA was fragmented into short fragments using fragmentation buffer and reverse transcribed into cDNA with random primers. Second-strand cDNA were synthesized by DNA polymerase I, RNase H, dNTP and buffer. The cDNA fragments were purified with QIAquick PCR extraction kit (Qiagen, Venlo, The Netherlands), end repaired, with poly (A) added, and then ligated to Illumina sequencing adapters. The ligation products were size selected by agarose gel electrophoresis, PCR amplified, and sequenced using Illumina HiSeq2500 by Gene Denovo Biotechnology Co (Guangzhou, China).

### Reads mapping and annotations

Quality reads of the raw RNA-Seq data were processed by FASTP (version 0.18.0) [23]. Short reads alignment tool Bowtie2 (version 2.2.8) was used for mapping reads to ribosome RNA (rRNA) database [24]. The remaining clean reads were further used for assembly. The index of reference genome was established according to the genomic data of perennial ryegrass [25]. The paired-end clean reads were mapped to the reference genome using HISAT 2.2.4 with “-rna-strandness RF” with other parameter settings as a default [26].

### Differential expression genes (DEGs) identification

StringTie v1.3.1 was employed to count the number of reads mapped to each gene and quantify the gene expression level in number of fragments per kilobase of the transcript sequence per million base pairs sequenced (FPKM) [27, 28]. The differential expression analysis was performed by DESeq2 [29] software between two different groups. The genes/transcripts with the parameter of false discovery rate (FDR) below 0.05 and absolute fold change ≥2 were considered DEGs/transcripts.

### Gene function annotation

Gene Ontology (GO) enrichment analysis provides all GO terms that are significantly enriched in DEGs comparing to the genome background and filters the DEGs that correspond to biological functions. Firstly, all DEGs were mapped to GO terms in the Gene Ontology database (http://www.geneontology.org/). The gene numbers were calculated for every term, and the significantly enriched GO terms in DEGs comparing to the genome background were defined by hypergeometric test. GO term with FDR ≤ 0.05 was considered significantly enriched by DEGs. The Kyoto Encyclopedia of Genes and Genomes (KEGG) is the major public pathway-related database [30]. KEGG pathway analysis was performed to retrieve the enriched pathway, using FDR ≤ 0.05 as a threshold for significantly enriched DEGs.

### WGCNA analysis, gene network co-expression and visualization

Weighted gene co-expression network analysis (WGCNA) is a systems biology method for describing the correlation patterns among genes across multiple samples. Co-expression networks were constructed using WGCNA (v1.47) package in R [31]. The selected 5862 DEGs were analyzed by WGCNA. The automatic network construction function blockwise Modules was performed to obtain modules with the default settings, except that the power is 8, with each module including at least 50 gene numbers. The networks were visualized using Cytoscape_3.3.0 [32].

### qRT-PCR analysis for target genes validation

Quantitative Real-time PCR (qRT-PCR) was used to verify the effectiveness of RNA-seq. Nine candidate genes were selected and verified by qRT-PCR, including *LOX2*, *HCAR*, *ACX*, *MFP*, *COL13*, *PER2*, *SODCP*, *HSP90–5* and *APX6*. RNA reverse transcription was performed using iScript™ cDNA (Bio-Rad Laboratories Inc.). Primers were designed with primer 3.0 [33] and is listed in Table S1. *eIF4A* was used as the reference gene to standardize the expression data [34]. The qRT-PCR reaction was carried out by using abm® EvaGreen 2X qPCR Master Mix (Applied Biological Materials Inc., Canada) and then performed at a fluorescence quantitative instrument (CFX-96; Bio-Rad). Three biological samples and three technical samples were used for all qRT-PCRs. The relative gene expression level was analyzed according to the 2^−ΔΔCt^ method [35].

## Results

### Identification of DEGs in different treatments

To investigate the possible regulatory mechanism of MeJA on heat responses of perennial, RNA-seq analysis was carried out on four different treatments. After the RNA-seq, a total of 583.2 million raw reads were generated from the 12 samples, ranging from 40.8 to 58.5 million for each sample. All raw transcriptome sequences were deposited in the National Center for Biotechnology Information (NCBI) sequence read archive (accession number: PRJNA766242). A total of 39.4 to 57.7 million clean reads were obtained after filtering, with the GC content between 52 and 54% and Q30>93% (Table S2). A total of 5504 new genes were identified (Table S3). There were 5862 DEGs analyzed in five comparison groups (CKvsT, CKvsH, CKvsT-H, TvsT-H and HvsT-H) (Table S4). CKvsT had the least DEGs, with 90 up-regulated genes and 28 down-regulated genes. CKvsH had the highest number of DEGs, including 2631 up-regulated genes and 1363 down-regulated genes. CKvsT-H and TvsT-H also had relatively more DEGs, containing 3639 and 3172 DEGs, respectively. The number of down-regulated DEGs in HvsT-H was more than that of up-regulated DEGs (Fig. S1).

### GO functional annotation and KEGG pathway analysis

**Fig. 1:**
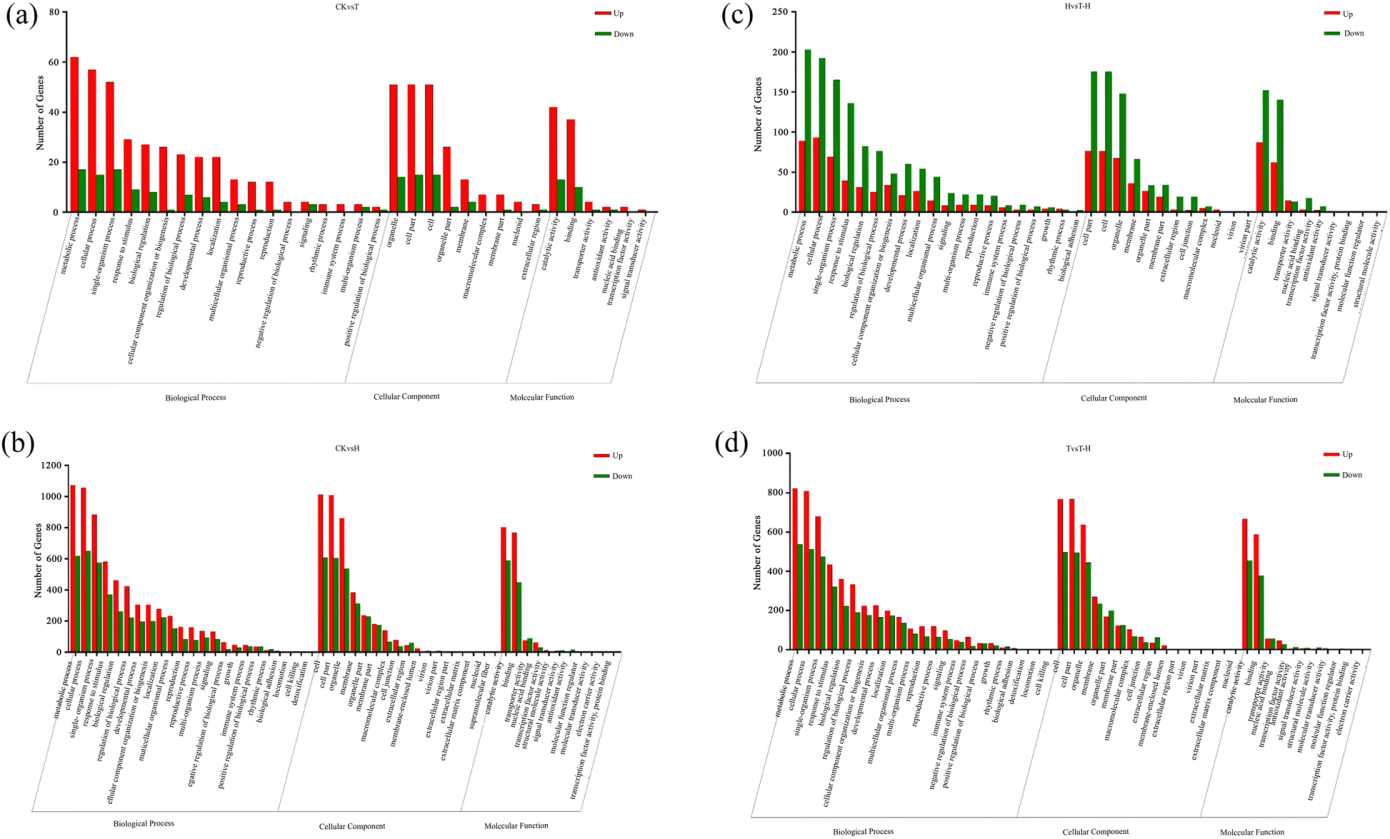
GO analysis of DEGs in different comparison groups in perennial ryegrass. **a**, CKvsT; **b**, CKvsH; **c**, HvsT-H; **d**, TvsT-H. CK, at cooler temperature without MeJA pretreatment; T, MeJA pretreatment at cooler temperature; H, high temperature at 38 °C; T-H, MeJA pretreatment and heat stress

The GO enrichment analysis of the 118 DEGs in CKvsT showed that the most abundant terms in the categories of biological processes were cellular process, metabolic process and single-organism process. Other most enriched categories were organelle, cell part and cell in the category of cell components and the catalytic activity and binding in the category of molecular function (Fig. 1a). Compared with CKvsT, CKvsH had more GO terms including cell killing, detoxification and signal transducer activity (Fig. 1b). Compared with CKvsT, HvsT-H had more terms such as growth, cell junction and transcription factor activity. Interestingly, HvsT-H had more number of down-regulated genes than up-regulated genes (Fig. 1c), which might be due to the altered expression of those genes in response to heat stress caused by MeJA pretreatment. The enrichment trend of up-regulated and down-regulated genes in DEGs of CKvsH and TvsT-H was similar (Fig. 1d), and these DEGs were most related to heat stress. In addition, the terms of CKvsT were mainly concentrated in the category of cellular components, most of which were related to plastid components, and most of these DEGs were up-regulated. (Fig. S2a, Table S5). CKvsH and TvsT-H shared many terms, mainly related to the photosynthetic system (Fig. S2b, d), and most of these DEGs were down-regulated. In addition, many DEGs related to the response of oxygen-containing compound were up-regulated in CKvsH and TvsT-H (Table S5). Most terms in HvsT-H were associated with stress response (Fig. S2c). The expression of DEGs related to the response to oxygen-containing compound was higher in CKvsH, and many genes related to the photosynthetic system were down-regulated. The expression of DEGs related to plastid components was higher in CKvsT.

**Fig. 2:**
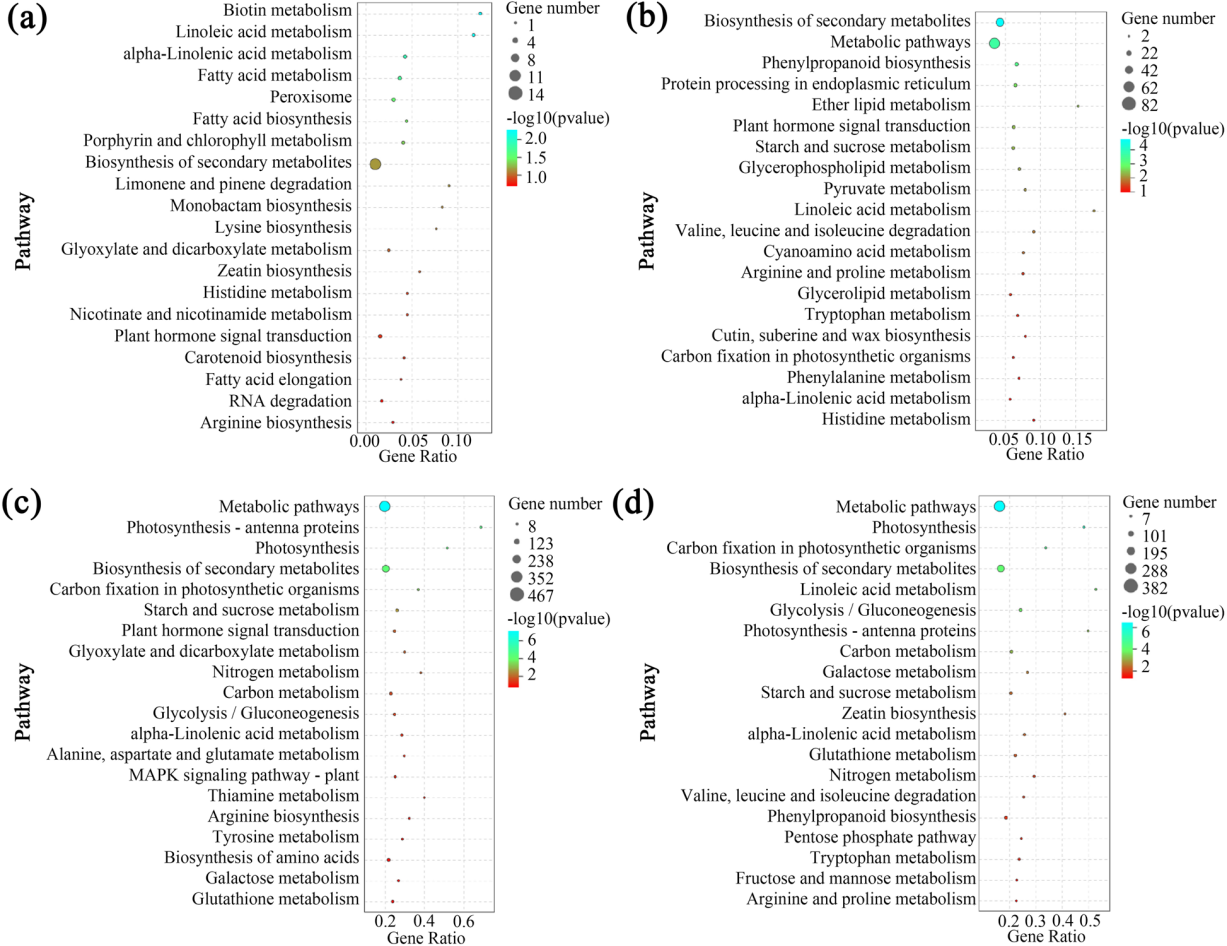
KEGG analysis of differential genes in different comparison groups of perennial ryegrass. **a**. CKvsT; **b**. CKvsH; **c**. HvsT-H; **d**. TvsT-H. Each figure showed top 20 pathways with Q values. CK, at cooler temperature without MeJA pretreatment; T, MeJA pretreatment at cooler temperature; H, high temperature at 38 °C; T-H, MeJA pretreatment and heat stress

KEGG analysis of the CKvsT showed that biosynthesis of secondary metabolites was the most enriched pathway mainly involved in biotin metabolism, fatty acid metabolism and peroxisome pathways (Fig. 2a). CKvsH was significantly enriched in some metabolic pathways, such as carbon metabolism and glutathione metabolism, as well as MAPK signaling pathway and amino acid biosynthesis (Fig. 2b). The most differentially enriched pathways in HvsT-H were protein processing in the endoplasmic reticulum, starch and sucrose metabolism, and pyruvate metabolism (Fig. 2c). The pathways enrichment of TvsT-H were mainly involved in various metabolic processes, including zeatin biosynthesis and phenylpropyl biosynthesis (Fig. 2d).

### Veen analysis of DEGs under CKvsH and TvsT-H

**Fig. 3:**
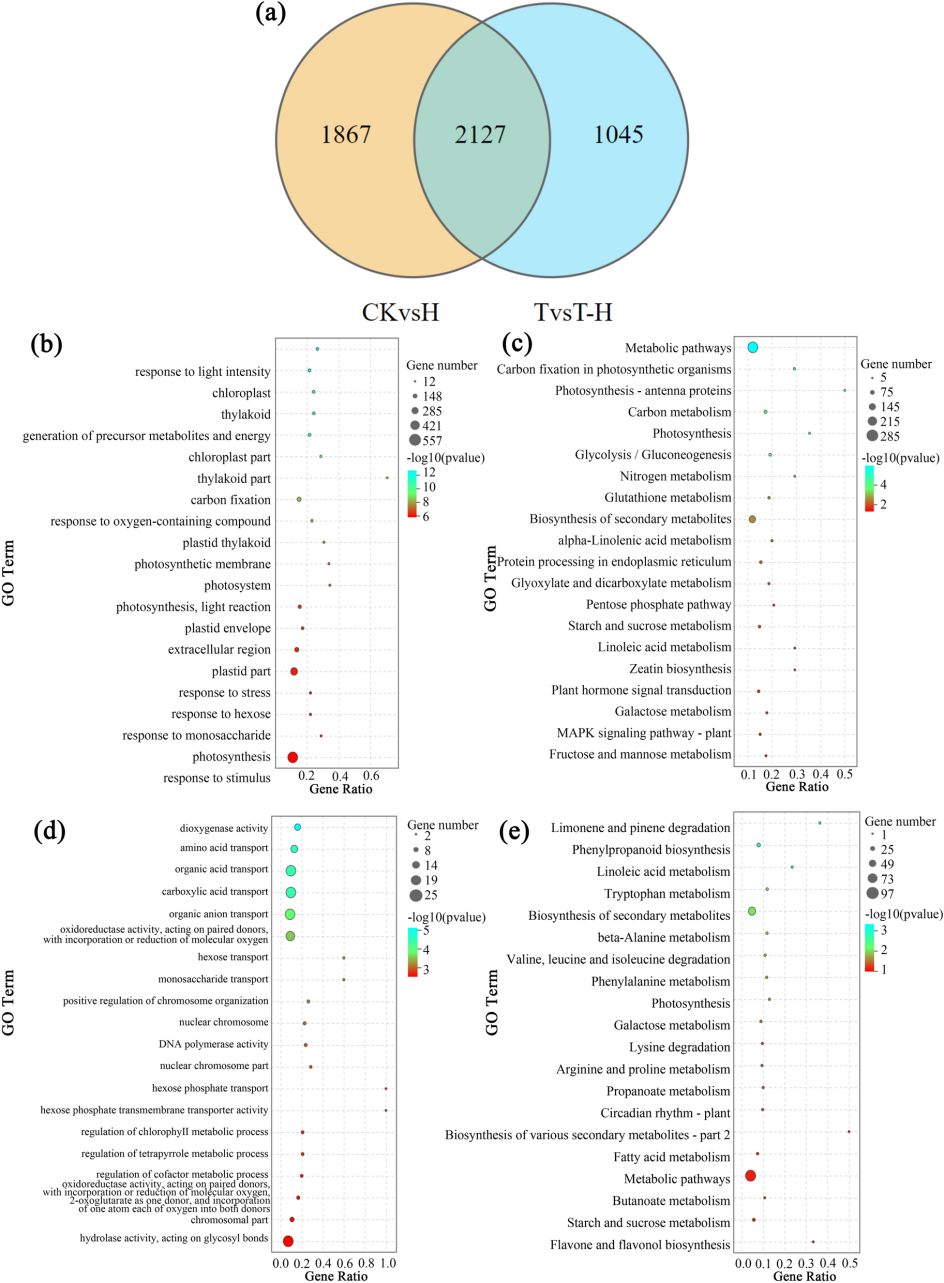
Comparative analysis of differences CKvsH and TvsT-H. **a** Veen of DEGs under CKvsH and TvsT-H. GO analysis and KEGG analysis of 2127 DEGs (**b**, **c**) and 1045 DEGs (**d**, **e**). CK, at cooler temperature without MeJA pretreatment; T, MeJA pretreatment at cooler temperature; H, high temperature at 38 °C; T-H, MeJA pretreatment and heat stress

A total of 2127 DEGs were shared between CKvsH and TvsT-H (Fig. 3a). These DEGs might be closely related to the response to heat stress. In the GO analysis, they were enriched in the terms of response to stimulus and stress (Fig. 3b). In KEGG pathway analysis, these DEGs were enriched in biosynthesis of secondary metabolites, photosynthesis-related pathways, Zeatin biosynthesis and MAPK signaling pathway (Fig. 3c). In both CKvsH and TvsT-H comparison groups, high temperature treatment resulted in more DEGs. In addition to shared DEGs, there were 1045 DEGs unique in TvsT-H, as result of the participation of MeJA (Fig. 3a). These genes were highly enriched in the terms of hydrolase activity, glycosyl bonds and many transportation-related. In addition, these DEGs were also enriched in the regulation of chlorophyll meta process and tetrapyrrole metabolic process (Fig. 3d). In KEGG analysis, these genes were mainly enriched in metabolic pathways, and also enriched in fatty acid metabolism, flavone and flavonol biosynthesis pathways (Fig. 3e).

### Co-expression network analysis

**Fig. 4:**
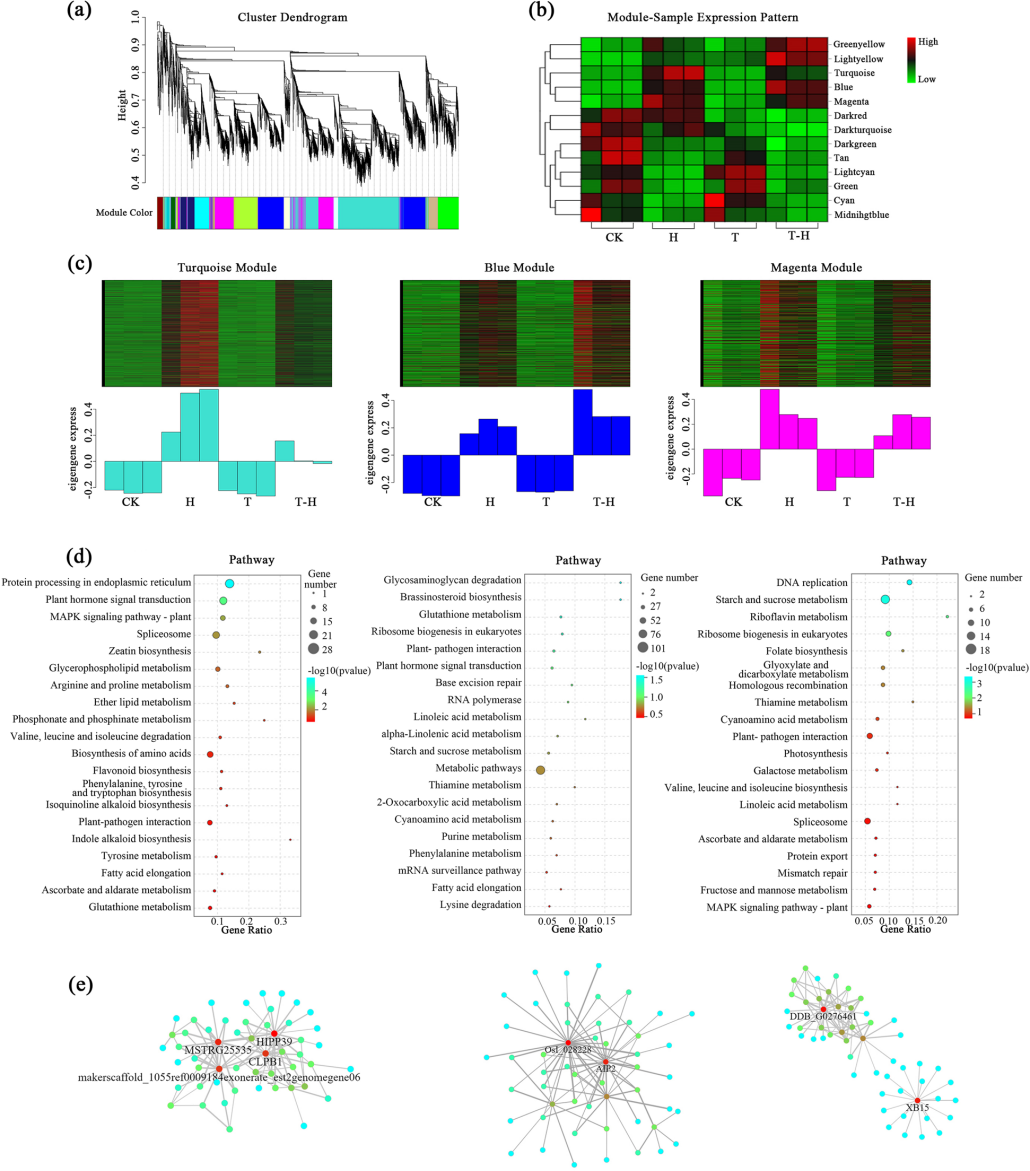
Weighted gene co-expression network (WGCNA) analysis of DEGs. **a** Cluster dendrogram; **b** Sample expression pattern; **c** Correlation between the top three gene modules with different number of genes and different treatments; **d** KEGG enrichment among the modules; (e) The correlation networks of hub genes corresponding to the modules

According to the clustering relationship between genes, 5862 DEGs were divided into 13 gene modules, with different color representing a specific module and containing a cluster of highly correlated genes (Fig. 4a, Fig. S3). The 13 modules had different associations with different samples (Fig. 4b). Of them, the “Turquoise”, “Blue” and “Magenta” modules covered 61.7% of DEGs (Fig. S3). The “Turquoise” module was specifically expressed in H treated samples, while the “Blue” and “Magenta” modules were expressed in both H and T-H treated samples (Fig. 4c). In the GO analysis, DEGs in the “Turquoise” module were mostly enriched in terms of stimulus response, chemical response and stress response (Fig. S4, Table S7), and in KEGG analysis, “Turquoise” module were significantly enriched in biosynthesis of amino acids pathway, protein processing in endoplasmic reticulum pathway and plant hormone signal transduction pathway (Fig. 4, Table S8). Most of the GO terms in the “Blue” module were related to nucleic acid binding and DNA binding, which was significantly enriched in the glycosaminoglycan degradation and the brassinosteroid biosynthesis pathways. The GO terms of “Magenta” module were mainly related to methylation and regulation of chromatin organization, and “Magenta” module was significantly enriched in the DNA replication and starch and sucrose metabolism pathways. It was also enriched in the photosynthetic pathway, ascorbate and aldarate metabolism pathway. The genes with the highest weight values were labeled in “Turquoise”, “Blue” and “Magenta” modules (Fig. 4). The top 10 kW_inthin_ value genes in the three modules were selected as candidate Hub genes, such as Zinc finger protein CONSTANS-LIKE 13 (*COL13*) and *HSP90–5* (Table S9).

### Analysis of candidate genes related to MeJA biosynthesis

**Fig. 5:**
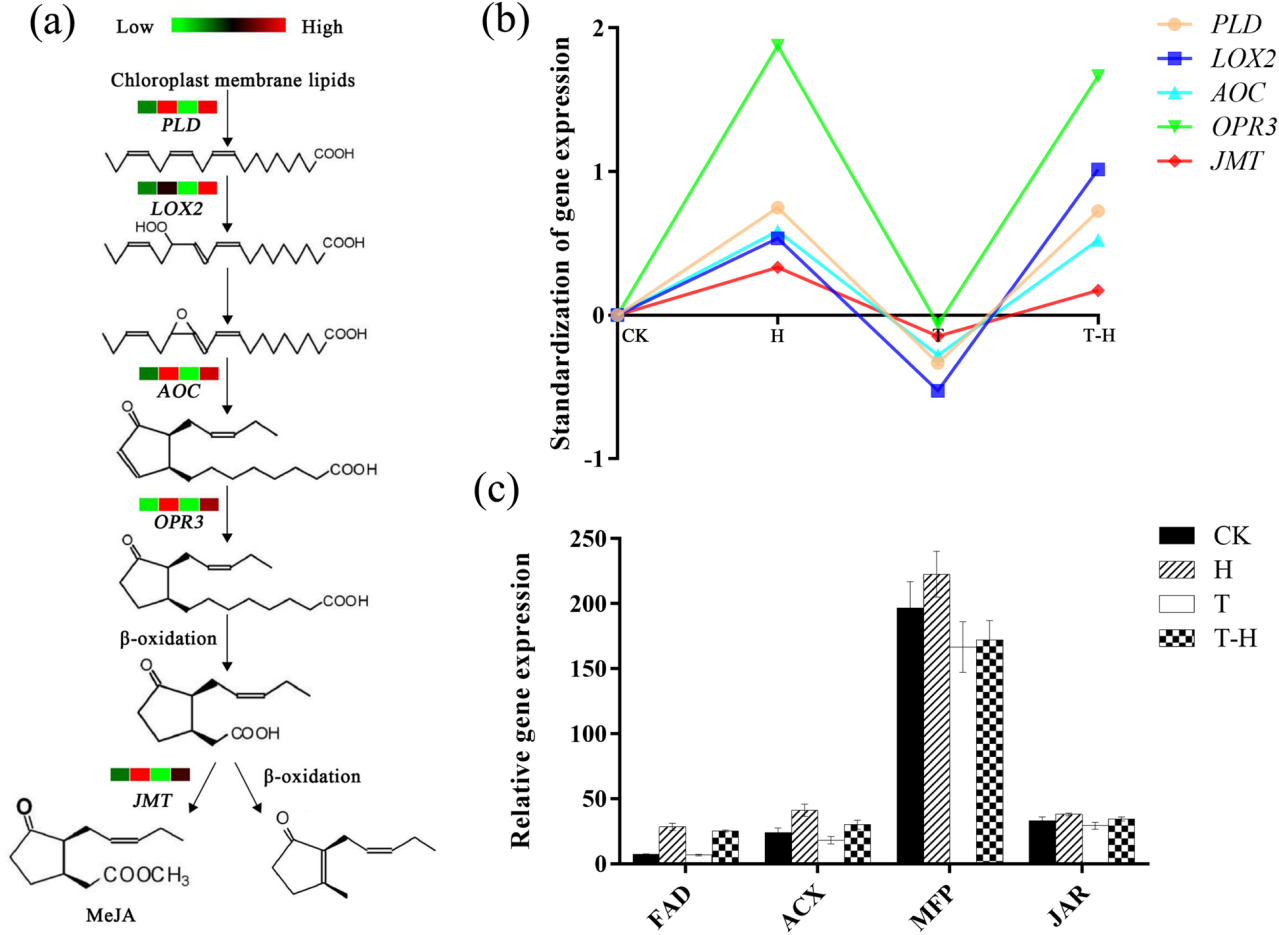
Analysis of gene expression related to the biosynthesis of MeJA. **a** Synthesis pathway of MeJA. Cubes represent to expression level in CK, H, T and T-H, with red color representing highest value and green color indicating the lowest; **b** Trend analysis of genes in the pathway under different treatments. These data were derived from RNA-seq, and normalization the data of different treatments with the control (CK). The abscissa from left to right are: CK, H, T, and T-H; **c** Expression analysis of other genes involved in the biosynthesis of MeJA. CK, at cooler temperature without MeJA pretreatment; T, MeJA pretreatment at cooler temperature; H, high temperature at 38 °C; T-H, MeJA pretreatment and heat stress

The biosynthesis of MeJA induced expression of phospholipase D (*PLD*), lipoxygenase (*LOX2*), allene oxide cyclase (*AOC*), 12-oxo-phytodienoic acid reductase (*OPR3*) and jasmonic acid carboxyl methyltransferase (*JMT*), and the expression levels of *PLD*, *LOX2*, *AOC*, *OPR3* and *JMT* were all higher in H and T-H than in CK (Fig. 5a). After data homogenization, the trend of gene expression under different treatments was compared with CK (Fig. 5b). Overall, the expressions of these five genes were up-regulated after H and T-H and down-regulated after T treatment. The upregulation of *LOX2* under T-H was greater than that under H, and the upregulation of *OPR3* under H and T-H was much higher than that of other genes (Fig. 5b). JAs are derived from a-linolenic acid, and the synthetic pathway from fatty acid to JAs also involves fatty acid desaturase (*FAD*), acyl-coa oxidase (*ACX*), multifunctional protein (*MFP*) and jasmonate resistant (*JAR*) [36]. The expression trends of these genes were similar under different treatments (Fig. 5c).

### The potential regulation of MeJA on chlorophyll metabolism under heat stress

**Fig. 6:**
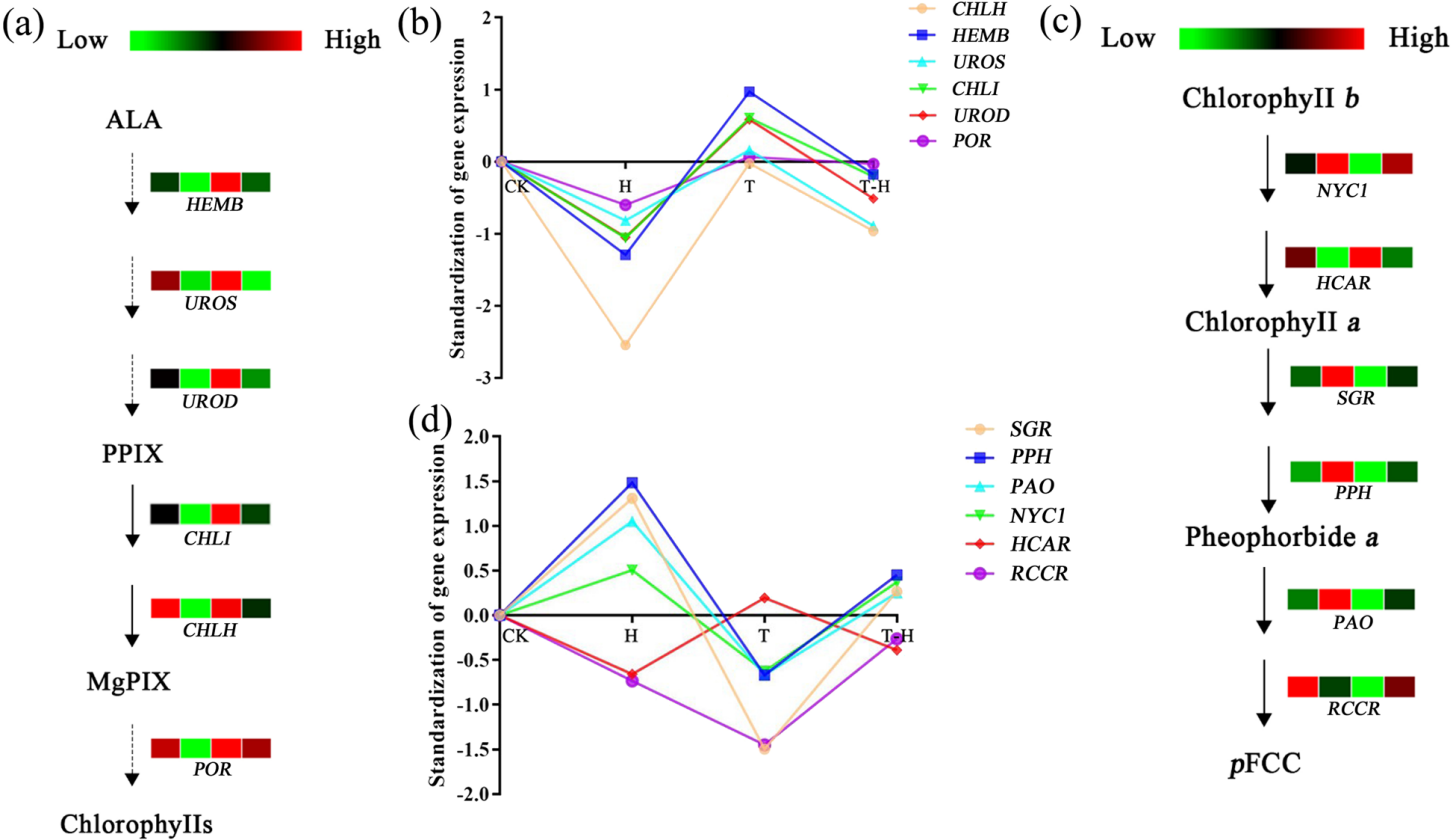
Expression analysis of genes related to chlorophyll biosynthesis and degradation pathway. **a** Chlorophyll synthesis pathway; **b** Trend analysis of related genes in chlorophyll biosynthesis pathway under different treatments. These data were derived from RNA-seq, and normalization the data of different treatments with the control (CK).; **c** Chlorophyll degradation pathway. In (a, c), cubes were representing to expression level in CK, H, T and T-H, with red color representing highest value and green color indicating the lowest; **d** Trend analysis of related genes in chlorophyll degradation pathway under different treatments. These data were derived from RNA-seq, and normalization the data of different treatments with the control (CK). In (**b**, **d**), the abscissa from left to right are: CK, H, T, and T-H. CK, at cooler temperature without MeJA pretreatment; T, MeJA pretreatment at cooler temperature; H, high temperature at 38 °C; T-H, MeJA pretreatment and heat stress

The chlorophyll content of plants usually decreases under stress conditions, which may be due to impaired biosynthesis or accelerated pigment degradation [37, 38]. Compared with CKvsH, the specific DEGs of TvsT-H were significantly enriched in the regulation of chlorophyll metabolism and tetrapyrrole metabolism (Fig. 3d). These results indicate that MeJA may be involved in chlorophyll related regulation during heat stress of perennial ryegrass. In this experiment, the expression levels of 5-aminolevulinate dehydratase (*HEMB*), uroporphyrinogen III synthase (*UROS*), uroporphyrinogen III decarboxylase (*UROD*), Mg-chelatase I-subunit (*CHLI*), Mg-chelatase H-subunit (*CHLH*) and protochlorophyllide oxidoreductase (*POR)* involved in the chlorophyll biosynthesis pathway were analyzed (Fig. 6a). Compared with CK, the expression of these genes was down-regulated after H treatment, while the expression of most of these genes was up-regulated only after T treatment. MeJA pretreatment had higher the expressions of most of these genes, compared to that of H but lower than that of CK (Fig. 6a, b). However, compared with CK, the expressions of non-yellow coloring 1 (*NYC1*), stay-green protein (*SGR*), pheophytin pheophorbide hydrolase (*PPH*) and pheophorbide a oxygenase (*PAO*) involved in the chlorophyll degradation pathway were up-regulated after H treatment, and their expressions in T-H treatment was lower than that in H. The expression trend of 7-hydroxy-chlorophyll a reductase (*HCAR*) was exactly opposite to that of other genes. Compared with CK, the expression of *HCAR* was down-regulated after H treatment, but MeJA pretreatment alleviated the down-regulation of this gene caused by high temperature. The expressions of red chlorophyll catabolite reductase (*RCCR*) gene in H, T, and T-H treatments were lower than that of CK (Fig. 6c, d).

### Expression analysis of antioxidant genes and HSF-HSPs networks

**Fig. 7:**
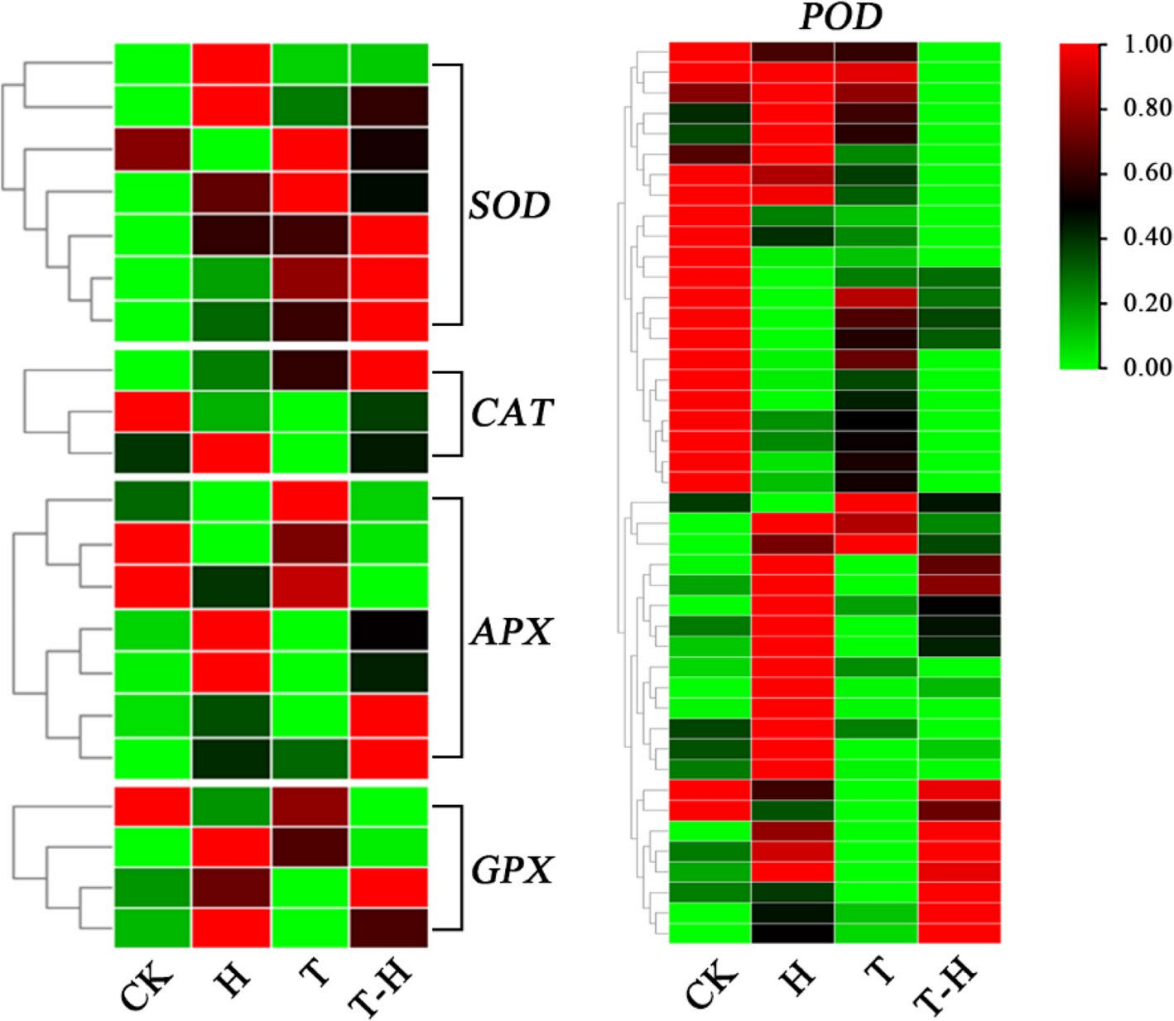
The expression of antioxidant genes under CK, H, T and T-H treatments, respectively. The genes in each box were annotated homologous genes. CK, at cooler temperature without MeJA pretreatment; T, MeJA pretreatment at cooler temperature; H, high temperature at 38 °C; T-H, MeJA pretreatment and heat stress

Plants produce a lot of reactive oxygen species (ROS) under high temperature stress [39, 40]. In the GO analysis of HvsT-H, it was found that the superoxide metabolic process was significantly enriched (Table S5). In order to explore whether MeJA would affect the antioxidant system of perennial ryegrass under heat stress, we analyzed the expression of different antioxidant enzyme genes. There were 21 DEGs related to antioxidant enzymes including superoxide dismutase (SOD), catalase (CAT), ascorbate peroxidase (APX) and glutathione peroxidase (GPX). Most of these genes were induced by heat stress, and some of them were expressed higher in T-H than in H treatment, such as plastidic Cu/Zn-SOD gene (*SODCP*), *APX2* and *GPX6*. Among the 44 peroxidase (POD)-related enzymes genes, nearly half of them had higher expressions in CK, while the others were mainly induced by heat stress (in H and T-H). Genes *PER70* and *PER21* were regulated by MeJA and under heat stress, but their expressions were decreased in T-H compared with H treatment (Fig. 7).

**Fig. 8:**
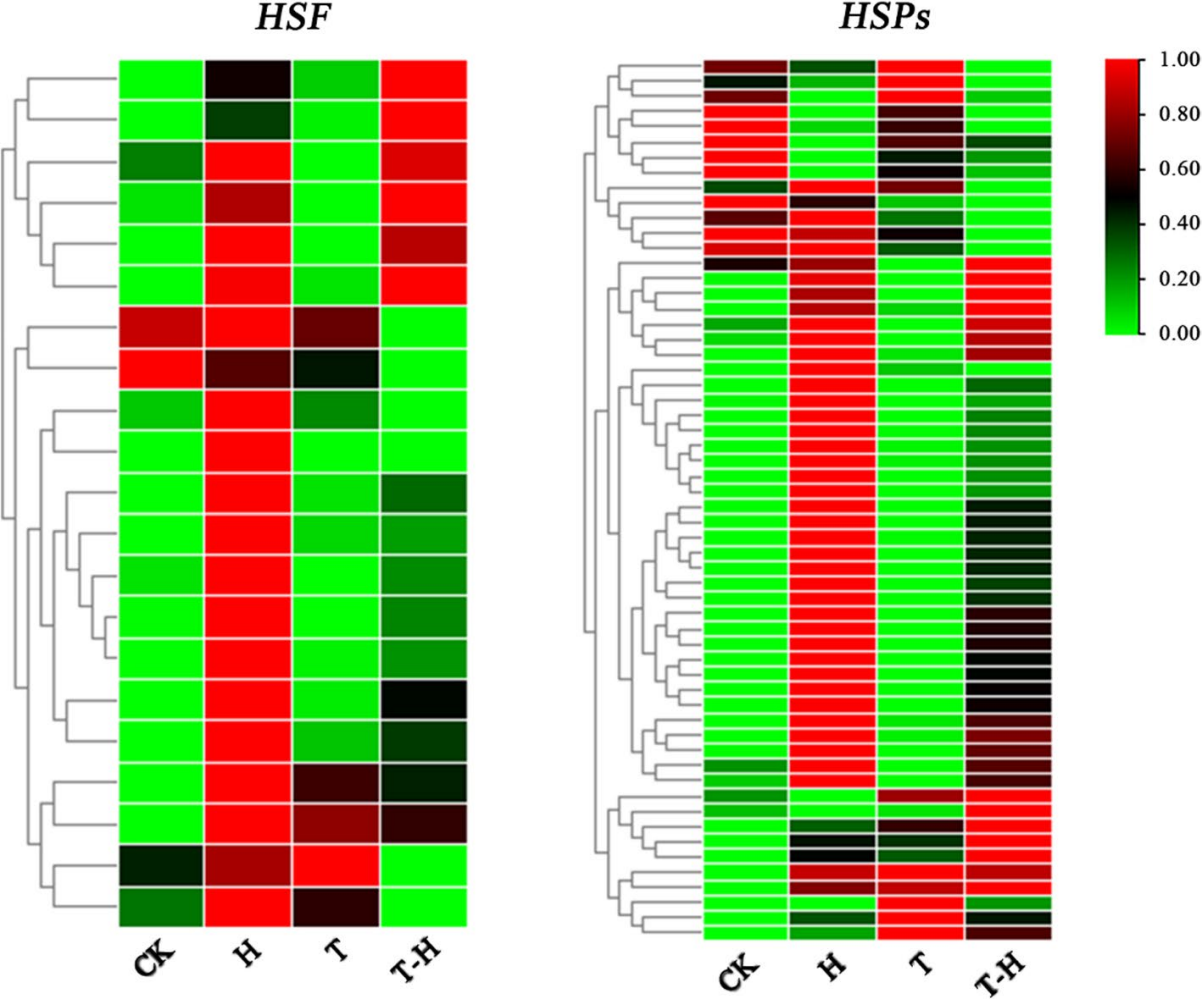
The expression of *HSF* and *HSPs* genes under CK, H, T and T-H treatments, respectively. CK, at cooler temperature without MeJA pretreatment; T, MeJA pretreatment at cooler temperature; H, high temperature at 38 °C; T-H, MeJA pretreatment and heat stress

Heat stress factors (HSF) and HSPs play key roles in plant heat tolerance [41]. Most *HSF* and *HSPs* genes were up-regulated under H, and some of them were down-regulated in T-H compared with H, such as *HSFB4C* and *HSP18*. There were also some genes showing the highest expression under T-H, such as *HSFA2C* and *HSP90*–5. These results indicated that MeJA had effects on the network regulation of HSF-HSPs in response to heat stress in perennial ryegrass (Fig. 8).

### qRT-PCR validation of the candidate genes expression profiles

**Fig. 9:**
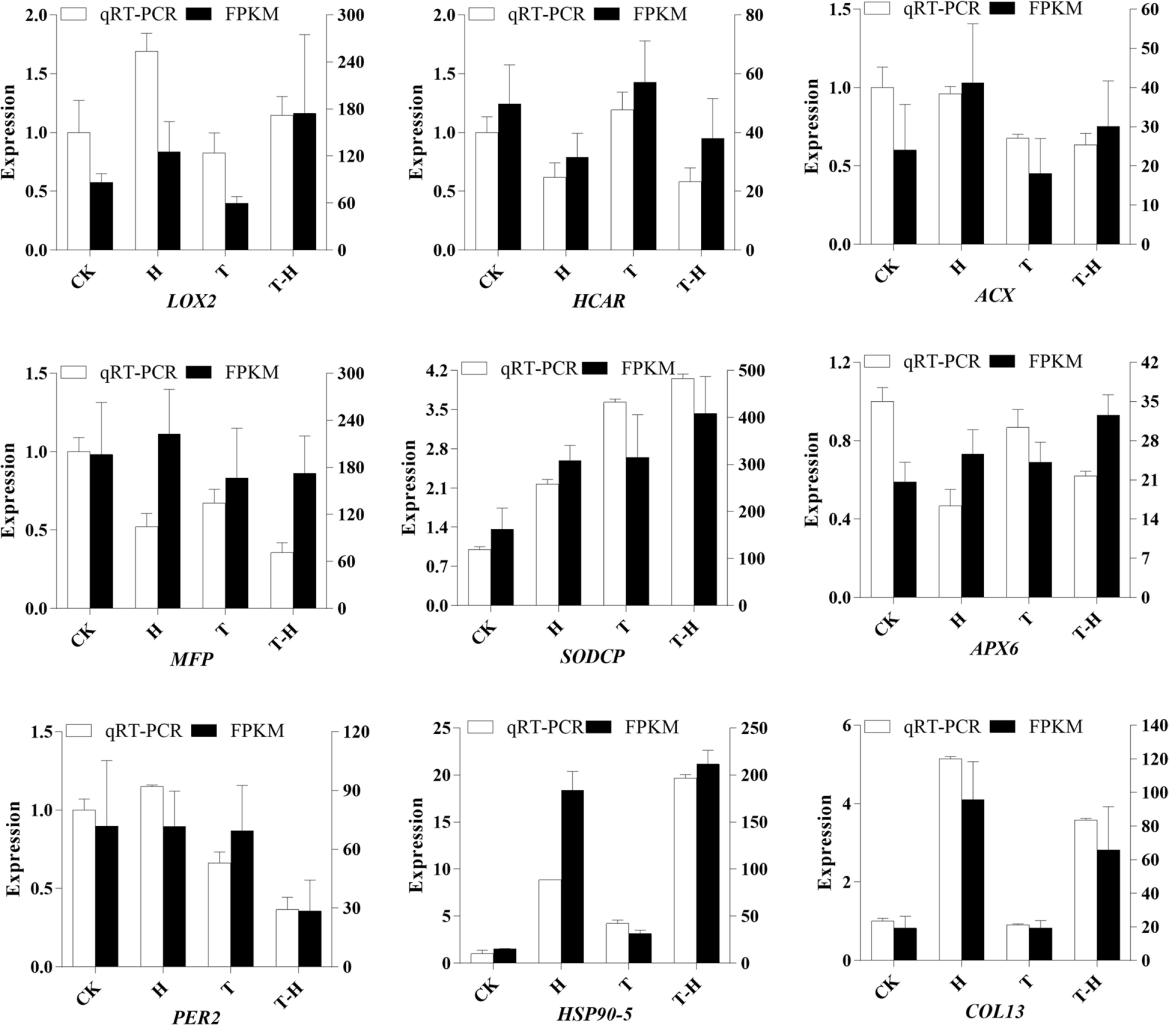
Expression profile of nine candidate genes by qRT-PCR and RNA-seq. Vertical bars indicate STDEV of each treatment. CK, at cooler temperature without MeJA pretreatment; T, MeJA pretreatment at cooler temperature; H, high temperature at 38 °C; T-H, MeJA pretreatment and heat stress

To verify the RNA-Seq results, nine genes were selected for qRT-PCR, including genes related to JAS biosynthesis, chlorophyll biosynthesis and degradation, peroxidase and module hub (Table S1). In general, the relative gene expression patterns were consistent with the sequencing results under different treatments (Fig. 9). For example, the expression level of *COL13* was the highest at H but lower in CK and T treatments. The expression of *SODCP* was the highest in T-H and lowest in CK. There were also a small number of genes which expression levels were slightly inconsistent with RNA-Seq trends. For example, the expression of *MFP* decreased in qRT-PCR but increased in the sequencing results under H.

## Discussion

Heat stress has a profound impact on plant growth and development, affecting about 2% of the plant genes [41]. Heat-stressed maize (*Zea Mays* L.) plants had significant changes in DEGs including *HSF*, *WRKY* and genes related to maintaining cell redox homeostasis such as *HSP* and antioxidant genes [42]. In rice (*Oryza sativa* L.), DEGs influenced by heat stress were significantly enriched in thylakoid and photosynthesis, especially photosynthesis-antenna proteins, carbon metabolism and photosynthesis pathways [43]. In this study, a total of 3994 DEGs were identified in the CKvsH comparison, which were also significantly enriched in photosynthetic system-related GO terms, carbon metabolism, plant hormone signal transduction and other pathways (Table S5, Fig. 2). JAs play an important role in mediating plant defense response to abiotic stress [44–46]. The regulation of JAs synthesis by exogenous MeJA is complex in plants, and most studies show that it is a positive feedback regulation on JAs biosynthesis [17, 47, 48]. It was reported that exogenous MeJA reduced the synthesis of endogenous JAs by inhibiting the activity of key enzymes (JA, MeJA, LOX, AOC and OPR) in citrus (*Citrus reticulata* Blanco.) [49], but application of MeJA increased the contents of endogenous JA and MeJA under heat stress in perennial ryegrass [17]. In this study, the expression level of JAs biosynthesis related genes decreased after addition of MeJA, which was similar to the results of Qiu et al (2020).

Heat stress negatively affects chlorophyll biosynthesis and photochemical reactions [50], while MeJA protects plants from photosynthetic damage [51]. Exogenous MeJA alleviated the decrease of chlorophyll content in soybean (*Glycine max* (Linn.) Merr.) and cowpea (*Vigna unguiculata* L.) under salt stress [52, 53]. Exogenous application of MeJA was beneficial to maintain the stability of chlorophyll content in perennial ryegrass leaves under heat stress [17]. In this study, a total of 1045 unique DEGs in TvsT-H were mainly enriched in the regulation pathway of chlorophyll metabolism (Fig. 3). When plants were subjected to heat stress, Chl biosynthesis was weakened, but degradation was accelerated [54]. Under heat stress, the expressions of five genes involved in chlorophyll biosynthesis decreased, but MeJA pretreatment alleviated the down-regulation of these genes. In addition, MeJA pretreatment reduced the expression level of chlorophyll degradation related genes. Previous study found that the expression levels of several Chl catabolic genes in heat sensitive ryegrass were significantly higher than that in heat resistant ryegrass under heat stress [5]. It appears that exogenous MeJA may delay leaf senescence in perennial ryegrass by affecting the expression level of genes in the chlorophyll biosynthesis and degradation pathways under heat stress, thus, improving the heat tolerance of perennial ryegrass.

Heat stress results in the production of a large number of reactive oxygen species (ROS) [41]. The overexpression of reactive oxygen scavenging enzymes such as SOD, CAT, APX and GPX improved plant stress resistance [55]. Exogenous application of MeJA protected cell membranes from heat stress by increasing the activity of antioxidant enzymes and improved heat resistance of *Arabidopsis* [16]. In this study, *APX2*, *CAT1*, and *SODCP* were highly expressed in T-H, and were strongly induced by MeJA under heat stress. Previous study proved that the overexpression of *SOD* and *APX* genes in transgenic potato enhanced its tolerance to heat and oxidative stress [56]. Therefore, results in this study indicated that MeJA improves the heat tolerance of perennial ryegrass by enhancing the expression of antioxidant genes. HSF-HSP networks are the central components of *Arabidopsis thaliana* heat shock regulatory network [57]. MeJA pretreatment had a significant effect on gene expression involved in HSF-HSP network. MeJA was down-regulated *CaHsfA6a* in pepper (*Capsicum annuum* L.) [58] and was induced *CaHsfB2a* under heat stress [59]. MeJA and heat shock treatment induced transcription of low-molecular-weight HSP genes (LMW HSPs) in *Pseudotsuga menziesii* (Mirb.) Franco. seedlings [60]. Wang et al. [61] found that HSFs responded strongly to heat stress, and the target genes of HSFs, such as *HSP* and *APX* showed strong and unique responses to different stressors in perennial ryegrass. In this study, in addition to the antioxidant genes, MeJA also induced the expression of *HSFA2C*, *HSP90–5* and *HSP80–1* under heat stress, and these genes may be important candidate genes for future heat resistant regulatory network study in this species. In general, MeJA was involved in the regulation of HSF-HSP network under heat stress, but the specific mechanism of molecular regulation by MeJA remained unclear. MeJA pretreatment could improve the heat resistance of perennial ryegrass, which may effectively improve the application of perennial ryegrass in practical production. Although Wang et al. [61] analyzed the response of *HSF* and its targets to heat stress in perennial ryegrass, the mechanism of exogenous MeJA participating in heat stress response in this study would laid new insight for heat tolerance genetic improvement of perennial ryegrass in the future.

## Conclusions

**Fig. 10:**
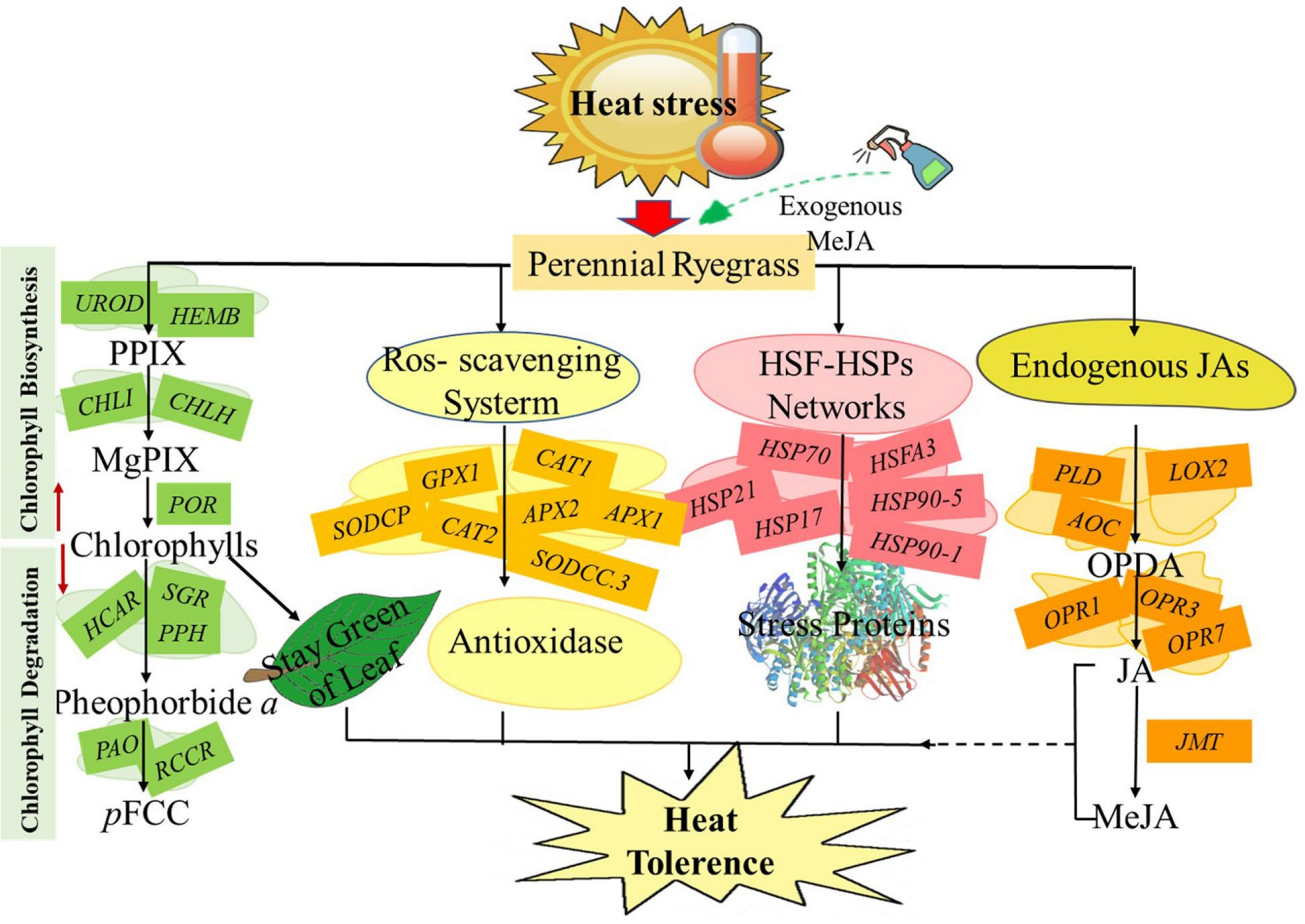
MeJA participates in the response of perennial ryegrass to heat stress. The red arrow with upward direction represented the positive correlations; the red arrow with downward direction represented the negative correlations; the black dotted line indicated that the direct correlation was uncertain

Heat stress adversely affects the growth and development of perennial ryegrass, and exogenous MeJA could improve its heat resistance. Compared with CKvsH, DEGs specific to TvsT-H was significantly enriched in chlorophyll metabolism related regulation. Exogenous MeJA increased the expression of genes involved in chlorophyll biosynthesis (*CHLH*, *HEMB*, *CHLI*) and decreased chlorophyll degradation genes (*SGR*, *PPH*), which contributed to stay green of plant leaf under heat stress. Superoxidation metabolism was significantly enriched in HvsT-H, and gene expression analysis showed that MeJA application enhanced the expression of antioxidant genes (*SODCP*, *APX2*), which alleviated oxidative damage. HSF-HSP network is an important way of heat stress response. MeJA participated in HSF-HSP network under heat stress, and enhanced the expression of genes (*HSFA2C*, *HSP90*–5, *HSP80*–1). The accumulation of stress protein developed a self-protective signal transduction pathway to ensure protection of unfolded protein. The results in this study elucidated that exogenous application of MeJA improved the heat tolerance of perennial ryegrass by mediating expression of genes in different pathways, such as chlorophyll biosynthesis and degradation, antioxidant enzyme system, HSF-HSP network and JAs biosynthesis. The transcriptome characterization of candidate genes for heat tolerance in perennial ryegrass after exogenous MeJA application is outlined as Fig. 10. Although important regulatory pathways of exogenous MeJA involved in heat response were identified in this study, candidate genes need to be further verified for their function.

## Supplementary Information

**Additional file 1: Table S1.** Primers list used in qRT-PCR. **Table S2**. Summary of the sequence clean data. **Table S3**. Reference comparison and gene statistics. **Table S4.** Statistical analysis of DEG in all samples. **Table S5.** Top 20 GO terms with Q values. **Table S6.** GO term shared by CKvsH and TvsT-H in the top 20 GO terms. **Table S7.** Top20 GO enrichment of three modules. **Table S8.** Top20 pathway of three modules. **Table S9.** The hub genes of three modules

**Additional file 2: Fig. S1.** The differentially expressed genes (DEGs) from 5 different comparisons. **Fig. S2.** Top 20 GO terms with Q values. **Fig. S3.** Module information of WGCNA. **Fig. S4.** Top 20 GO terms with Q values of the three modules

## Data Availability

Sequencing data for perennial ryegrass was available on NCBI (PRJNA766242).
