# Supplementary material for: Transcriptome characterization of candidate genes for heat tolerance in perennial ryegrass after exogenous methyl Jasmonate application
Source: BMC Plant Biol. 2022 Feb 12;22:68. doi: 10.1186/s12870-021-03412-9 (PMC8840555; doi:10.1186/s12870-021-03412-9)
Supplement: Supplementary file 2 — Additional file 2: Fig. S1. The differentially expressed genes (DEGs) from 5 different comparisons. Fig. S2. Top 20 GO terms with Q values. Fig. S3. Module information of WGCNA. Fig. S4. Top 20 GO terms with Q values of the three modules [file 12870_2021_3412_MOESM2_ESM.docx]

Figure S1


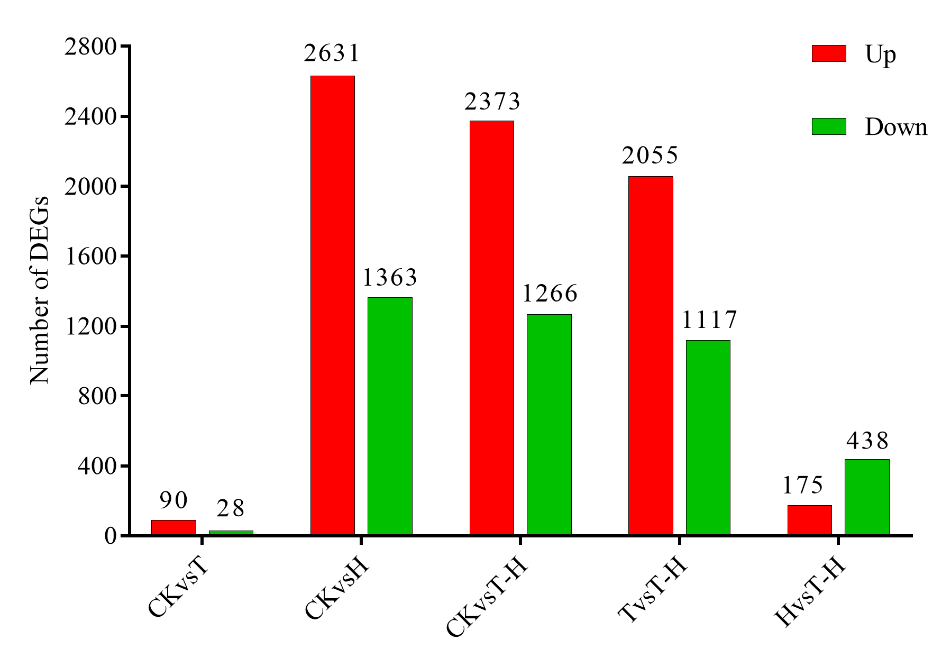


Figure S1 The differentially expressed genes (DEGs) from 5 different comparisons. CK, at cooler temperature without MeJA pretreatment; T, MeJA pretreatment at cooler temperature; H, high temperature at 38 °C; T-H, MeJA pretreatment and heat stress.

Figure S2


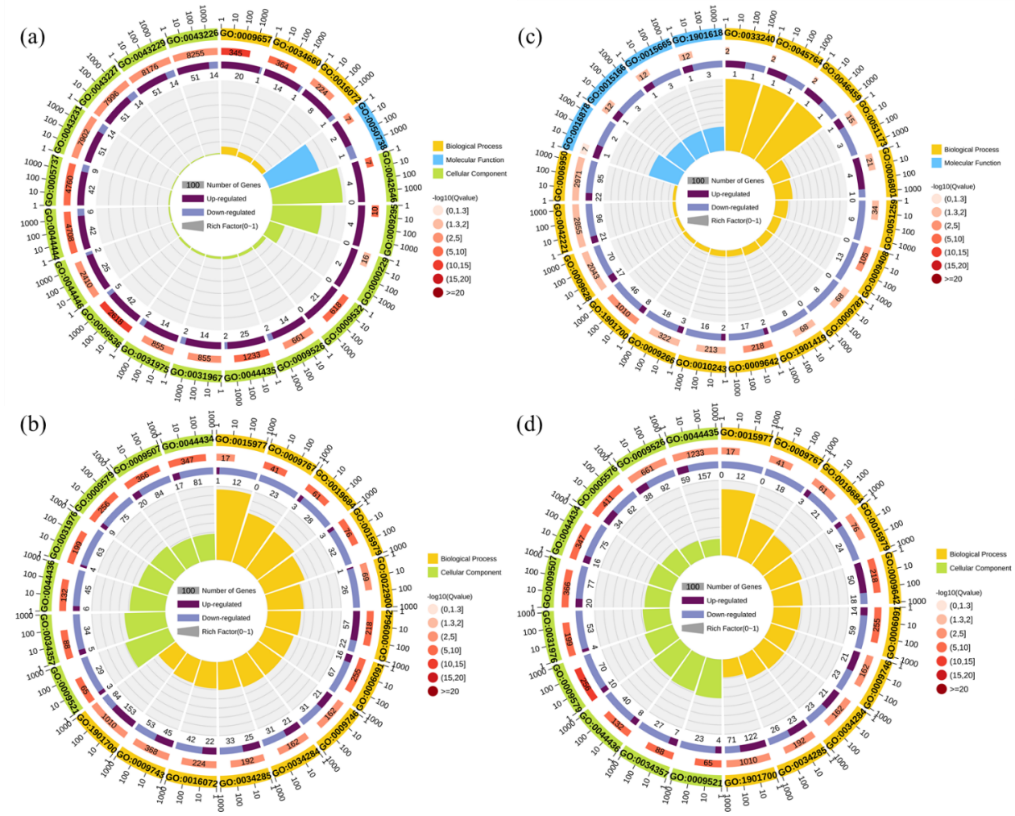


Figure S2 Top 20 GO terms with Q values. (a) Top 20 GO terms with Q values in CKvsT; (b) Top 20 GO terms with Q values in CKvsH; (c) Top 20 GO terms with Q values in HvsT-H; (d) Top 20 GO terms with Q values in TvsT-H. CK, at cooler temperature without MeJA pretreatment; T, MeJA pretreatment at cooler temperature; H, high temperature at 38°C; T-H, MeJA pretreatment and heat stress.

Figure S3


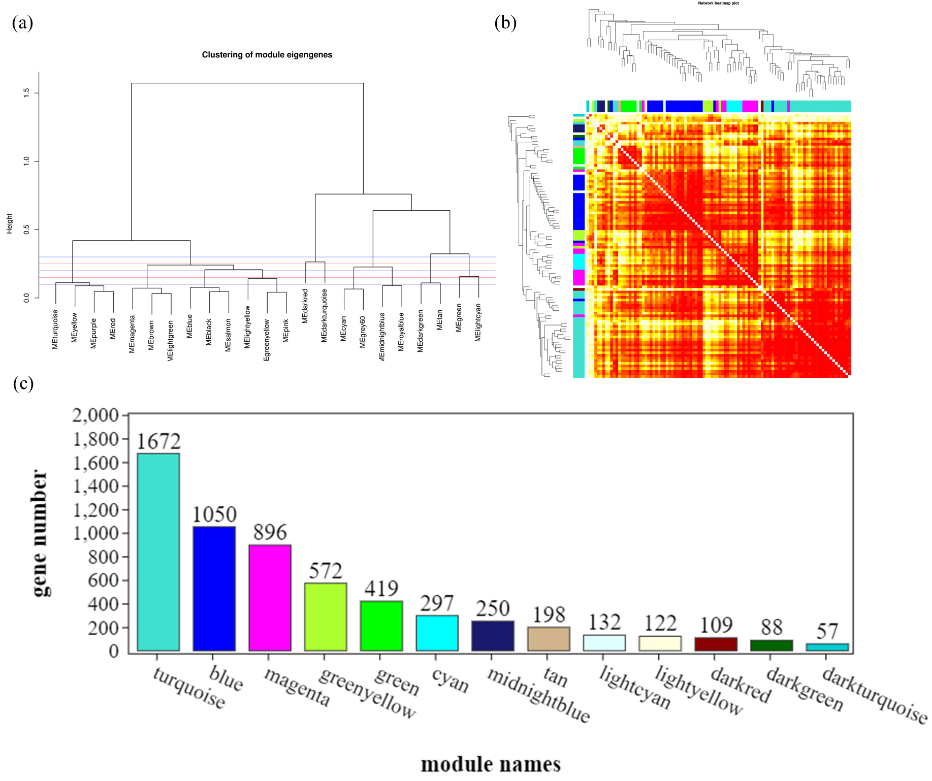


Figure S3 Module information of WGCNA. (a) Clustering of module eigengenes; (b) Modular gene correlation analysis; (c) Gene count statistics of each module. CK, at cooler temperature without MeJA pretreatment; T, MeJA pretreatment at cooler temperature; H, high temperature at 38 °C; T-H, MeJA pretreatment and heat stress.

Figure S4


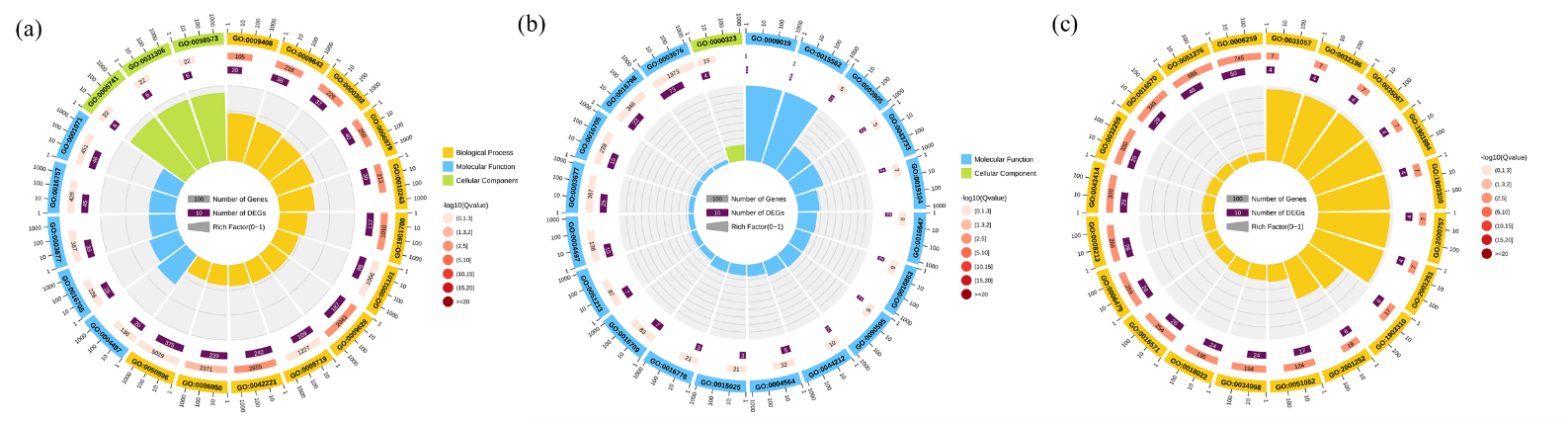


Figure S4 Top 20 GO terms with Q values of the three modules. a: The top 20 Go Enrichment in the "Turquoise" module; b: The top 20 Go Enrichment in the "Blue" module; c: The top 20 Go Enrichment in the "Magenta" module.
